# A New and Robust Prognostic Biomarker for Mortality Risk Prediction in Dialysis Patients With Coronary Artery Disease: Red Cell Distribution Width-to-Albumin Ratio

**DOI:** 10.31083/RCM47939

**Published:** 2026-04-20

**Authors:** Xuecheng Zhao, Enmin Xie, Zhengqin Zhai, Di Sun, Siqi Shen, Haoming He, Wei Liu, Lin Cai, Hesong Zeng, Jingang Zheng

**Affiliations:** ^1^Department of Cardiology, China–Japan Friendship Hospital, Affiliated With Capital Medical University, 100029 Beijing, China; ^2^Department of Cardiology, Fuwai Hospital, National Center for Cardiovascular Diseases, Chinese Academy of Medical Sciences and Peking Union Medical College, 100037 Beijing, China; ^3^Department of Cardiology, Chengdu Third People's Hospital, 610031 Chengdu, Sichuan, China; ^4^Department of Cardiology, Tongji Hospital, Tongji Medical College, Huazhong University of Science and Technology, 430030 Wuhan, Hubei, China

**Keywords:** coronary artery disease, dialysis, red cell distribution width, albumin, red cell distribution width-to-albumin ratio

## Abstract

**Background:**

Coronary artery disease (CAD) remains a fundamental etiology of morbidity and mortality among patients receiving dialysis, a cohort known to have a particularly poor prognosis. While traditional CAD risk factors, such as hyperlipidemia, hypertension, and diabetes, remain noteworthy, these factors do not fully capture the complexity of the disease in dialysis patients. Therefore, innovative and robust biomarkers that can refine risk stratification and enhance prognostic precision in this vulnerable population are imperative. Hence, this study aimed to evaluate the use of the red cell distribution width-to-albumin ratio (RAR) to predict clinical outcomes, particularly in dialysis patients with CAD.

**Methods:**

We analyzed data from a multicenter cohort of 1128 CAD patients on dialysis who were enrolled between January 2015 and June 2021. Patient stratification into tertiles was performed based on RARs. The primary endpoints were all-cause mortality and cardiovascular mortality. The secondary endpoint was the occurrence of major adverse cardiovascular events (MACEs), comprising non-fatal myocardial infarction, non-fatal stroke, and other cardiovascular events.

**Results:**

The median follow-up duration was 20.9 months 378 (33.5%) patients experienced all-cause mortality, 261 (19.1%) cardiovascular mortality, and 485 (43.0%) MACEs. In multivariable Cox proportional hazards regression, patients in the highest RAR tertile demonstrated remarkably escalated risks of all-cause mortality (hazard ratio (HR): 1.592, 95% confidence interval (CI): 1.212–2.092), cardiovascular mortality (HR: 1.503, 95% CI: 1.086–2.080), and MACEs (HR: 1.452, 95% CI: 1.141–1.846) compared with those in the lowest tertile. Moreover, restricted cubic spline analysis revealed a linear, dose-dependent association between elevated RAR and an increased risk of these adverse outcomes. The integration of the RAR into existing risk models, specifically the Global Registry of Acute Coronary Events and Gensini scores, noticeably amplified the predictive performance, as demonstrated by notable enhancements in both net reclassification improvement and integrated discrimination improvement.

**Conclusions:**

An elevated RAR serves as an independent predictor of poor outcomes in patients with CAD undergoing dialysis.

**Clinical Trial Registration:**

This study was registered at ClinicalTrials.gov (Identifier: NCT05841082; https://clinicaltrials.gov/study/NCT05841082).

## 1. Introduction

On a global scale, estimates indicate that 5 to 7 million patients have end-stage renal disease (ESRD), a critical condition necessitating renal replacement therapies, particularly dialysis [1, 2]. Coronary artery disease (CAD) constitutes a predominant component of cardiovascular pathology in this patient population, with a staggering prevalence of 42%, a rate that is several times higher than that identified in patients without renal failure [3, 4]. ESRD patients are not only more likely to experience multivessel coronary artery involvement, but they also present with more advanced stages of the disease and remarkably worse clinical outcomes relative to individuals with intact renal function [4]. Hence, traditional risk factors for CAD, particularly those related to lipid metabolism, undergo notable alterations in ESRD, with dysregulated lipid profiles and an accelerated progression of atherosclerosis [5]. Moreover, the pathogenesis of CAD in ESRD is further complicated by the presence of non-traditional risk factors, including accumulation of uremic toxins, endothelial dysfunction, vascular calcification, and chronic inflammatory states, all of which significantly amplify the cardiovascular burden in such patients [6]. These multifactorial challenges emphasize the necessity of identifying and validating innovative prognostic markers, providing more precise risk stratification and prognostic insights for ESRD patients who also suffer from CAD.

Among various hematologic indices, the red blood cell distribution width (RDW), a measure of the variability in red blood cell (RBC) size, has garnered clinicians’ attention as a potential marker of erythrocyte heterogeneity and an indirect indicator of systemic inflammation [7]. Although RDW has traditionally been employed in the differential diagnosis of anemia, an elevation of this biomarker in the context of ESRD has emerged as a robust and independent prognostic indicator. Increased RDW has been consistently associated with adverse clinical outcomes, such as escalated mortality and cardiovascular events [8]. Furthermore, hypoalbuminemia is a marker of both mortality and morbidity in patients undergoing dialysis, with lower albumin levels being associated with elevated cardiovascular risk and poorer overall prognosis [9, 10, 11, 12]. The red cell distribution width-to-albumin ratio (RAR) is an innovative biomarker of systemic inflammation and oxidative stress in ESRD patients [13]. However, data on the association of RAR with long-term cardiovascular outcomes, including all-cause mortality, cardiovascular mortality and major adverse cardiovascular events (MACEs), remain limited in patients with ESRD and concomitant CAD.

In this study, we aimed to elucidate the link between RARs and key clinical endpoints, including all-cause mortality, cardiovascular mortality, and MACEs, in a cohort of dialysis patients with CAD. Moreover, we attempted to identify the incremental prognostic value of integrating the RAR into established clinical risk scoring systems, including the Global Registry of Acute Coronary Events (GRACE) and Gensini scores, to determine whether this novel biomarker improves cardiovascular risk prediction. This study was performed based on data acquired from a large, multicenter cohort in China.

## 2. Materials and Methods

### 2.1 Study Design and Population

We utilized data from the CRUISE-R cohort (Coronary Revascularization in Patients on Dialysis in China-Retrospective; NCT05841082), a robust multicenter, observational registry designed to investigate the clinical features of CAD patients undergoing dialysis in China. The study was performed under strict adherence to the ethical standards established by the Declaration of Helsinki, and approved by the Ethics Committee of China–Japan Friendship Hospital (authorization number 2022-KY-075-1). Given the study’s non-invasive design and the stringent protocols implemented to maintain patient anonymity, the requirement for informed consent was waived.

Inclusion criteria were as follows: (1) age between 18 and 80 years, inclusive; (2) receipt of long-term dialysis, including either peritoneal dialysis or hemodialysis, with a dialysis duration of at least 3 months and regular dialysis sessions performed at least twice per week; and (3) angiographically confirmed CAD, defined as ≥50% luminal stenosis in at least one coronary artery, including ST-segment elevation myocardial infarction, non–ST-segment elevation acute coronary syndrome, and stable coronary artery disease.

Exclusion criteria included: (1) patients who were not receiving dialysis or had a dialysis duration of less than 3 months; (2) absence of significant coronary artery stenosis on coronary angiography; (3) coronary angiography performed for indications unrelated to CAD; (4) repeat hospital admissions, with only data from the first admission included in the analysis; and (5) incomplete or missing data on red cell distribution width or serum albumin levels.

**Fig. 1.**
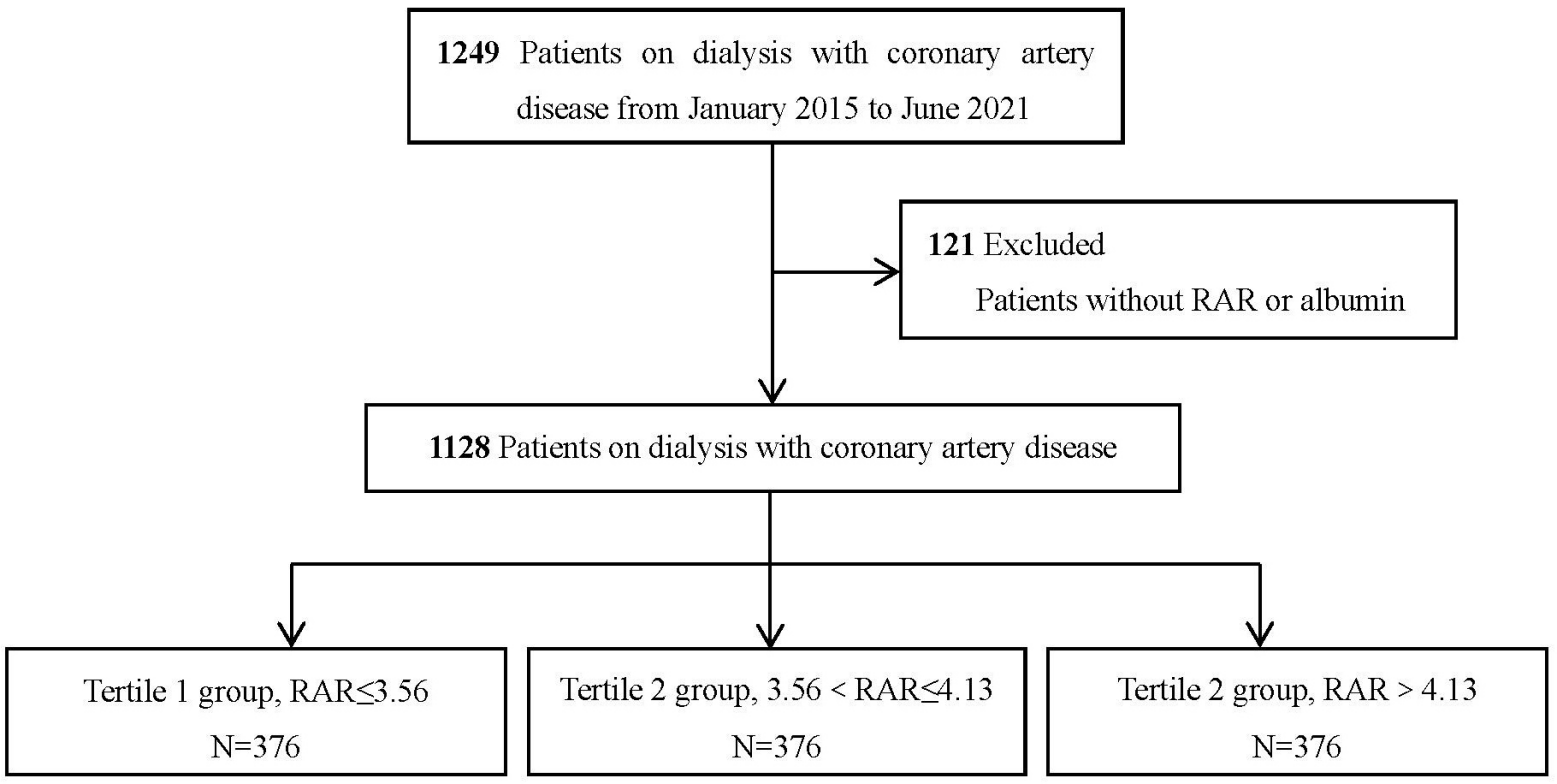
**Flowchart of the patient selection process**. RAR, red cell distribution width-to-albumin ratio.

A thorough review of 455,617 cardiac catheterization records from 30 tertiary medical centers was undertaken across 12 provinces in mainland China, covering the period from January 2015 to June 2021 (**Supplementary Table 1**). Patients with a dialysis duration of fewer than 3 months or not undergoing dialysis were excluded (n = 453,421). Patients without significant coronary artery stenosis evident on angiography (n = 328), as well as those who underwent angiography for indications unrelated to CAD were excluded (n = 87). Additionally, subsequent admissions were excluded (n = 532). This rigorous selection process led to 1249 eligible CAD patients who were on dialysis. After further excluding 121 patients with missing or incomplete data on red cell distribution width or serum albumin levels, 1128 patients remained for the final analysis. These patients were categorized into three groups of equal size (n = 376) according to tertiles of RAR: Tertile 1 (RAR ≤3.56), Tertile 2 (3.56 < RAR ≤ 4.13), and Tertile 3 (RAR >4.13) (Fig. 1).

### 2.2 Data Collection and Definitions

We acquired data from electronic medical records. The dataset involved a comprehensive array of variables, comprising demographic features, comorbid conditions, cardiac function parameters, CAD severity, procedural and therapeutic interventions, as well as clinical outcomes. Dialysis-specific information was also thoroughly documented, comprising details on dialysis modality, treatment duration, and the underlying indication for dialysis. Laboratory test results obtained upon admission, prior to the initiation of any therapeutic interventions, involved fundamental biomarkers (e.g., hemoglobin, creatinine, and lipid profiles). To ensure the accuracy of the data, principal variables, such as diagnoses, dialysis-related parameters, and clinical outcomes were entered independently by two trained researchers, and any discrepancies between their entries were resolved by a third reviewer.

The RDW was calculated as the ratio of the standard deviation of the red blood cell size to the mean cell volume, multiplied by 100. The reference range of the RDW was 11.5–15.5%. The RAR was subsequently derived by dividing the RDW (%) by the serum albumin concentration (g/dL). CAD severity was obtained via the established GRACE and Gensini scoring systems, alongside the presence of multivessel coronary artery involvement (**Supplementary Table 2**). These assessments were implemented with the assistance of two blinded interventional cardiologists.

Given the unique characteristics of the dialysis population, the measurement methods for RDW and their reference ranges may vary compared with those established for the general population. In this study, we ensured that the reference range for RDW (11.5–15.5%) and the detection methodology were validated and adjusted specifically for the dialysis cohort to account for potential deviations in RDW resulting from dialysis-related factors such as altered fluid balance and erythropoiesis. These adjustments aimed to ensure the reliability of RDW as a biomarker in this population. The timing of albumin testing can significantly affect the accuracy of the measurements, especially in patients undergoing dialysis who may experience fluctuating levels of albumin due to dialysis procedures. Albumin levels were measured upon admission, prior to any therapeutic interventions or dialysis treatments. This timepoint was selected to minimize the impact of albumin shifts induced by dialysis, ensuring that the recorded values reflect the patient’s baseline status rather than post-dialysis fluctuations.

### 2.3 Follow-Up

All-cause mortality and cardiovascular mortality were the primary endpoints, both of which were robustly tracked. Secondary endpoints involved MACEs, e.g., non-fatal myocardial infarction, non-fatal stroke, and other cardiovascular events. The specific definitions for these outcomes are accessible in **Supplementary Table 3**. Follow-up data collection was implemented through a combination of telephone interviews and reviews of outpatient clinic records, with the follow-up period extending until June 30, 2022. In the case of multiple clinical events, only the first event was included in the outcome analysis. Patients with unavailable follow-up data were censored at the time of their last known clinical contact to minimize potential bias related to incomplete outcome data.

### 2.4 Statistical Analysis

Statistical analyses were performed using R software (version 4.2.2) (https://cran.r-project.org/bin/windows/base/old/4.2.2/R-4.2.2-win.exe), and a *p*-value < 0.05 was considered statistically significant. Continuous variables were expressed as the mean (± standard deviation) or median (interquartile range [IQR]), depending on the underlying data distribution. Differences between groups were evaluated using analysis of variance (ANOVA) or the Kruskal-Wallis test, as appropriate based on data normality. Categorical variables were expressed as counts and percentages, and group differences were examined using the Chi-square or the Fisher’s exact tests.

Kaplan-Meier survival curves were drawn to investigate time-to-event outcomes, with differences between curves assessed using the log-rank test to clarify the association between the RAR and event-free survival. To further investigate the link between RAR and clinical outcomes, we employed both univariate and multivariate Cox proportional hazards regression models. Restricted cubic splines (RCS) with four knots were used to explore nonlinear associations between the RAR and clinical outcomes.

The ability of the RAR to amplify the predictive accuracy of the GRACE and Gensini scores was determined through two widely accepted metrics, including continuous net reclassification improvement (NRI) and integrated discrimination improvement (IDI). To account for potential confounders related to RDW detection methods and albumin levels, sensitivity analyses were performed.

Subgroup analyses were implemented to determine whether associations between the RAR and clinical outcomes remained consistent across patient subgroups. To ensure the validity of the Cox proportional hazards model, we assessed the proportional hazards assumption using the Schoenfeld residuals test. The test was conducted for each of the models in our analysis.

The goodness of fit for all statistical models, including the Cox proportional hazards regression models and RCS analysis, was assessed using the Akaike Information Criterion (AIC) and Bayesian Information Criterion (BIC). These criteria were employed to ensure the models’ adequacy and to select the most parsimonious model that best fits the data. For the RCS analysis, four knots were selected based on clinical relevance and optimal fit as determined by AIC/BIC values. The models’ goodness of fit was evaluated using the likelihood ratio test. Interaction *p*-values were computed using likelihood ratio tests, and interaction terms with *p*-values < 0.05 were considered statistically significant.

## 3. Results

### 3.1 Baseline Characteristics

Overall, 1128 CAD patients undergoing dialysis were involved. The participants’ baseline demographic and clinical features, stratified by the RAR, are outlined in Table 1. The study cohort was predominantly male, constituting approximately 75% of the population, with a mean age of 62 years. The most frequent comorbidities were hypertension and diabetes mellitus. The median RAR of the entire cohort was 3.83.

**Table 1.** **Clinical characteristics of patients at baseline stratified by tertiles of the RAR**.

			Overall	Tertile 1	Tertile 2	Tertile 3	*p*-value
			n = 1128	n = 376	n = 376	n = 376	
Age (years), median (IQR)			62.00 (55.00–69.00)	61.00 (54.00–68.00)	61.00 (55.00–69.00)	64.00 (56.75–72.00)	<0.001
Male, No. (%)			835 (74.0)	283 (75.3)	280 (74.5)	272 (72.3)	0.639
Medical history and risk factors, No. (%)							
	Hypertension		1048 (92.9)	352 (93.6)	344 (91.5)	352 (93.6)	0.423
	Diabetes mellitus		597 (52.9)	195 (51.9)	208 (55.3)	193 (51.3)	0.556
	Heart failure		70 (6.2)	18 (4.8)	29 (7.7)	23 (6.1)	0.250
	Atrial fibrillation		102 (9.0)	23 (6.1)	37 (9.8)	42 (11.2)	0.043
	Valvular disease		36 (3.2)	8 (2.1)	13 (3.5)	15 (4.0)	0.327
	Cerebrovascular disease		208 (18.4)	65 (17.3)	69 (18.4)	74 (19.7)	0.698
	Peripheral arterial disease		105 (9.3)	25 (6.6)	32 (8.5)	48 (12.8)	0.013
	Previous myocardial infarction		139 (12.3)	39 (10.4)	39 (10.4)	61 (16.2)	0.019
Previous intervention, No. (%)							
	PCI		209 (18.5)	69 (18.4)	65 (17.3)	75 (19.9)	0.640
	CABG		17 (1.5)	7 (1.9)	5 (1.3)	5 (1.3)	0.787
Dialysis status							
	Dialysis modality, No. (%)						<0.001
		Hemodialysis	1027 (91.0)	368 (97.9)	348 (92.6)	311 (82.7)	
		Peritoneal dialysis	101 (9.0)	8 (2.1)	28 (7.4)	65 (17.3)	
	Vintage, years		3.00 (1.33–6.00)	3.04 (1.42–6.00)	3.17 (1.73–6.00)	2.96 (1.00–5.08)	0.005
	Cause of dialysis, No. (%)						0.003
		Diabetes mellitus	344 (30.5)	95 (25.3)	111 (29.5)	138 (36.7)	
		Others	784 (69.5)	281 (74.7)	265 (70.5)	238 (63.3)	
	Index presentation, No. (%)						0.011
		AMI	988 (87.6)	315 (83.8)	331 (88.0)	342 (91.0)	
		Non-AMI	140 (12.4)	34 (9.0)	45 (12.0)	61 (16.2)	
Laboratory Tests							
	RDW-CV, median (IQR)		14.40 (13.60–15.50)	13.70 (13.00–14.10)	14.40 (13.70–15.20)	15.60 (14.70–16.90)	<0.001
	Albumin (g/dL), median (IQR)		3.80 (3.46–4.12)	4.19 (3.98–4.42)	3.78 (3.60–4.00)	3.38 (3.08–3.60)	<0.001
	RAR, median (IQR)		3.83 (3.44–4.32)	3.31 (3.14–3.44)	3.83 (3.69–3.96)	4.59 (4.32–5.12)	<0.001
	Alkaline phosphatase (U/L), median (IQR)		82.00 (63.00–109.00)	81.50 (62.00–109.17)	81.90 (62.00–107.25)	84.00 (64.00–112.50)	0.597
	Hemoglobin (g/L), median (IQR)		105.00 (92.00–117.00)	110.00 (99.00–122.00)	104.50 (95.00–116.70)	98.00 (82.00–112.00)	<0.001
	TC (mmol/L), median (IQR)		3.72 (3.10–4.51)	3.74 (3.17–4.62)	3.76 (3.20–4.52)	3.57 (2.96–4.33)	0.004
	TG (mmol/L), median (IQR)		1.60 (1.11–2.35)	1.75 (1.21–2.64)	1.70 (1.17–2.34)	1.38 (0.98–2.00)	<0.001
	HDL-C (mmol/L), median (IQR)		0.89 (0.73–1.10)	0.91 (0.75–1.14)	0.85 (0.73–1.06)	0.90 (0.71–1.10)	0.028
	LDL-C (mmol/L), median (IQR)		2.12 (1.60–2.73)	2.13 (1.60–2.74)	2.19 (1.70–2.78)	2.02 (1.55–2.64)	0.099
	Glucose (mmol/L), median (IQR)		6.40 (4.82–9.27)	6.24 (4.83–9.03)	6.33 (4.72–8.98)	6.67 (5.02–9.61)	0.206
	Serum creatinine (mg/dL), median (IQR)		8.60 (6.70–10.80)	8.70 (6.75–10.65)	8.90 (7.00–11.30)	8.20 (6.30–10.60)	0.010
	LVEF, No. (%)		56.00 (45.00–62.00)	58.00 (47.00–64.00)	57.00 (47.00–63.00)	52.50 (40.75–60.00)	<0.001
Procedure characteristic, No. (%)							
	Radial access		865 (76.7)	307 (81.6)	280 (74.5)	278 (73.9)	0.020
	Any left main disease		119 (10.5)	41 (10.9)	43 (11.4)	35 (9.3)	0.614
	Multi-vessel disease (≥2 vessels)		944 (83.7)	317 (84.3)	307 (81.6)	320 (85.1)	0.405
	PCI treatment		795 (70.5)	271 (72.1)	258 (68.6)	266 (70.7)	0.577
	GRACE score		156.00 (133.00–180.00)	144.00 (126.75–171.00)	153.50 (130.75–176.25)	168.50 (144.00–189.25)	<0.001
	Gensini score		52.00 (32.00–81.00)	44.00 (28.00–72.00)	50.50 (31.00–81.00)	56.00 (34.00–86.00)	<0.001
Discharge medications, No. (%)							
	Dual antiplatelet therapy		154 (13.7)	49 (13.0)	52 (13.8)	53 (14.1)	0.907
	Aspirin		1033 (91.6)	350 (93.1)	341 (90.7)	342 (91.0)	0.432
	Clopidogrel		919 (81.5)	306 (81.4)	304 (80.9)	309 (82.2)	0.894
	Ticagrelor		124 (11.0)	40 (10.6)	46 (12.2)	38 (10.1)	0.624
	ACE inhibitor or ARB		521 (46.2)	172 (45.7)	184 (48.9)	165 (43.9)	0.372
	β-blocker		906 (80.3)	303 (80.6)	297 (79.0)	306 (81.4)	0.702
	Statin		1064 (94.3)	358 (95.2)	349 (92.8)	357 (94.9)	0.298

Tablenotes: Data are presented as mean (SD), median (IQR), or n (%). IQR, interquartile range; PCI, percutaneous coronary intervention; CABG, Coronary Artery Bypass Grafting; AMI, acute myocardial infarction; RDW-CV, red blood cell distribution width - coefficient of variation; RAR, red cell distribution width-to-albumin ratio; TC, Total Cholesterol; TG, Triglyceride; HDL-C, High-Density Lipoprotein Cholesterol; LDL-C, Low-Density Lipoprotein Cholesterol; LVEF, Left Ventricular Ejection Fraction; GRACE, Global Registry of Acute Coronary Events; SD, Standard Deviation.

The Tertile 3 group demonstrated a distinct distribution with a higher proportion of patients undergoing peritoneal dialysis and fewer patients on hemodialysis relative to the other tertiles, although the majority of the cohort (91.0%) was receiving hemodialysis. In addition, the Tertile 3 group had a relatively shorter duration of dialysis. Diabetes was the predominant indication for renal replacement therapy in all groups. Patients in Tertile 3 were older and had a higher prevalence of peripheral artery disease, prior myocardial infarction, and atrial fibrillation. This group also exhibited escalated high-density lipoprotein cholesterol (HDL-C) levels and lower left ventricular ejection fraction (LVEF), hemoglobin, total cholesterol, and triglycerides. Furthermore, the Tertile 3 group included fewer patients undergoing radial artery access for coronary angiography and had higher Gensini and GRACE scores relative to the other two tertiles.

### 3.2 Outcomes

**Fig. 2.**
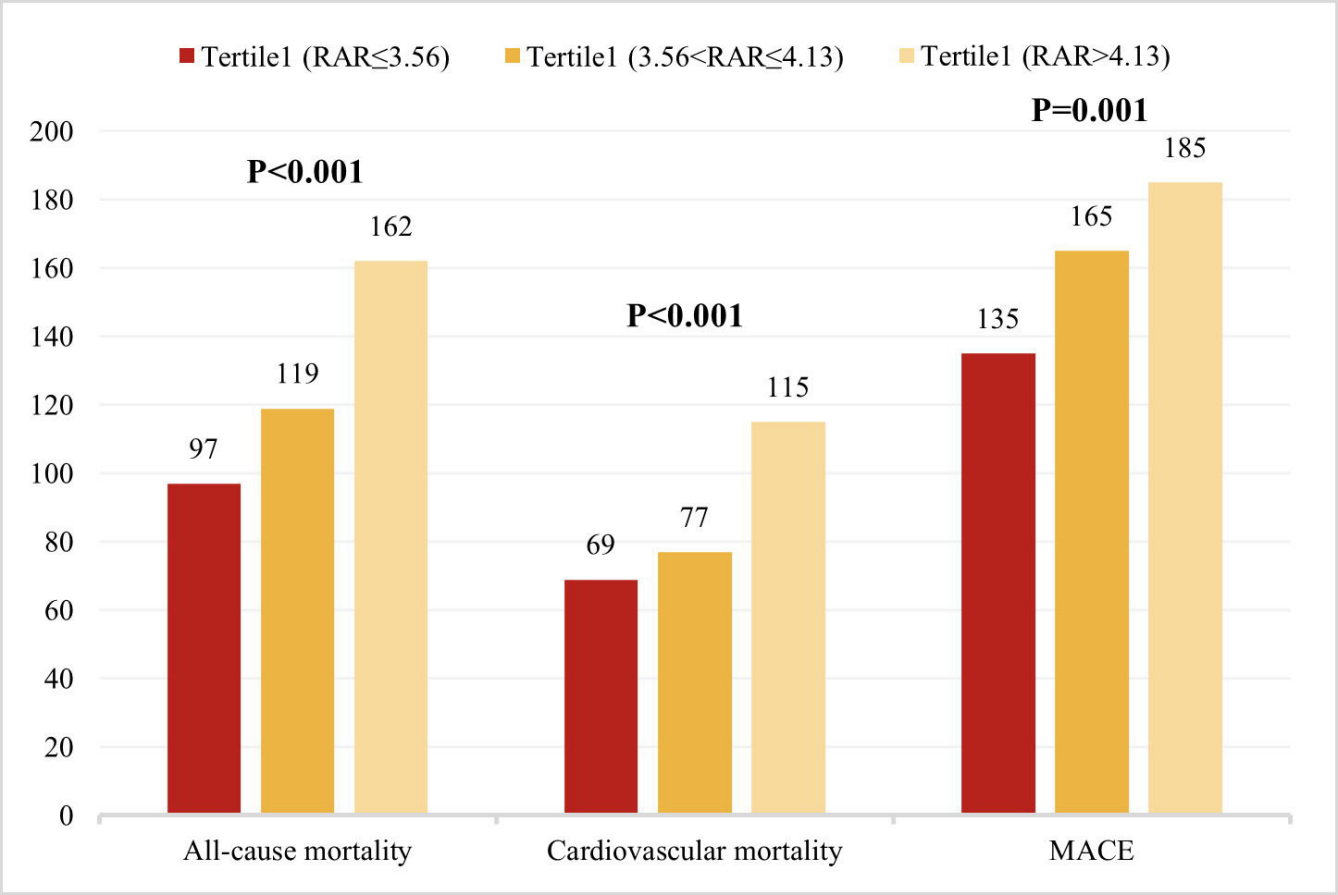
**Distribution of primary and key secondary outcome events**. Comparison of all-cause mortality, cardiovascular mortality, and MACEs across RAR tertiles.

The median follow-up duration was 20.9 months, with an IQR of 18–30 months. During this time frame, there were 378 (33.5%) cases of all-cause mortality and 261 (23.1%) cases of cardiovascular mortality. Overall, 485 (43.0%) MACEs were documented, comprising 111 non-fatal myocardial infarctions and 41 non-fatal strokes (**Supplementary Table 4**). The highest incidence rates of all-cause mortality, cardiovascular mortality, and MACEs were found in the Tertile 3 group, with all comparisons yielding *p*-values ≤ 0.001 (Fig. 2). The Schoenfeld residuals test for the proportional-hazards assumption confirmed that the Cox regression models used in this study were valid. No violations of the proportional-hazards assumption were observed (*p *= 0.11), ensuring the reliability of the hazard ratios (HRs) reported in our analysis.

**Fig. 3.**
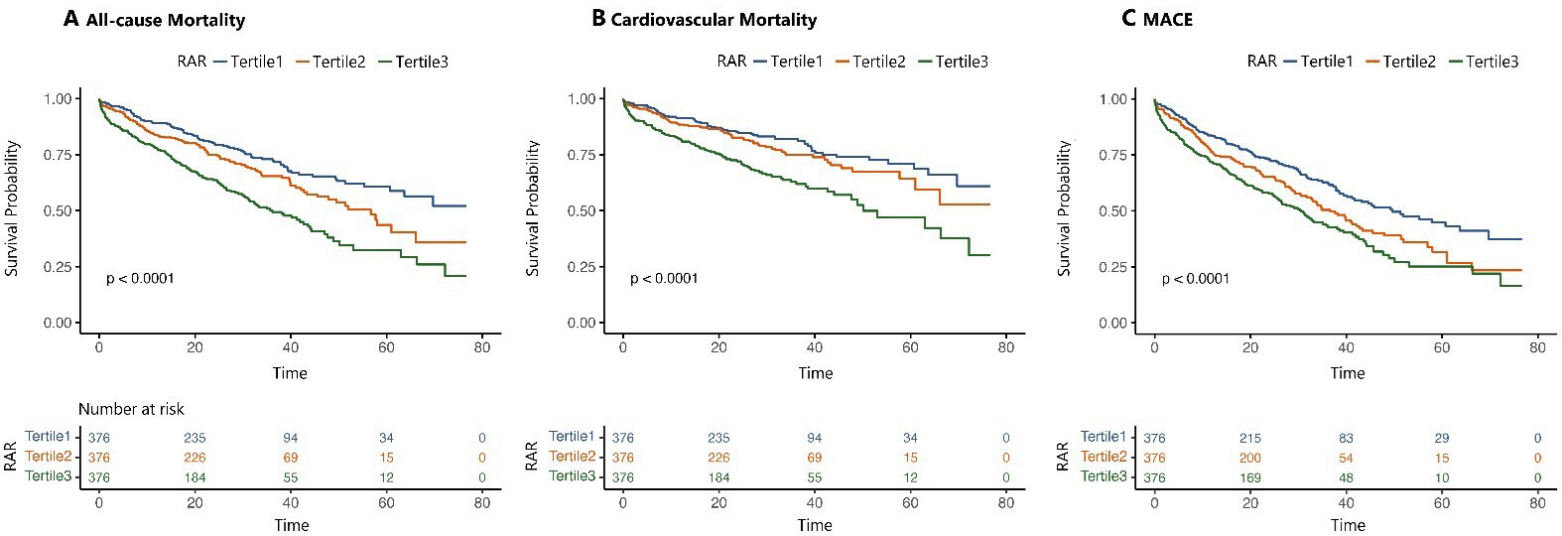
**Time-to-event analysis of primary and secondary outcomes by RAR tertiles**. (A,B) illustrate the time-to-event analysis of the primary outcomes: all-cause mortality and cardiovascular mortality, respectively. (C) Time-to-event analysis for the composite secondary endpoint: MACEs, major adverse cardiovascular events, including all-cause mortality, non-fatal myocardial infarction, and non-fatal stroke. The analysis was stratified by the three RAR tertiles.

Fig. 3 illustrates the outcomes across the RAR tertiles using Kaplan-Meier survival curves. The event rates for all-cause mortality, cardiovascular mortality, and MACEs increased progressively across RAR tertiles (*p* < 0.001 for each comparison). Table 2 outlines the association of the RAR tertiles with the primary and secondary endpoints. Moreover, univariate Cox regression analysis (Model 1) revealed a remarkable and progressive elevation in the risk of all-cause mortality, cardiovascular mortality, and MACEs with increasing RAR tertiles. This positive link persisted after adjusting for demographic features, namely age and sex (Model 2). In Model 3, after adjusting for a comprehensive array of procedural interventions, dialysis-related variables, and clinical risk factors, the highest RAR tertile remained an independent predictor of elevated risk of all-cause mortality (HR: 1.592, 95% confidence interval (CI): 1.212–2.092), cardiovascular mortality (HR: 1.503, 95% CI: 1.086–2.080), and MACEs (HR: 1.452, 95% CI: 1.141–1.846) relative to the reference group.

**Table 2.** **Associations between the RAR and primary and key secondary outcomes**.

		Model 1			Model 2			Model 3		
		HR	95% CI	*p*	HR	95% CI	*p*	HR	95% CI	*p*
All-cause mortality^1^										
	RAR^4^	1.384	1.257–1.523	0.000	1.336	1.209–1.477	0.000	1.229	1.101–1.372	0.000
	RAR1	ref.	ref.	ref.	ref.	ref.	ref.	ref.	ref.	ref.
	RAR2	1.379	1.054–1.804	0.019	1.325	1.013–1.733	0.04	1.296	0.988–1.702	0.061
	RAR3	2.193	1.703–2.824	0.000	1.959	1.518–2.528	0.000	1.592	1.212–2.092	0.001
Cardiovascular mortality^2^										
	RAR^4^	1.362	1.212–1.531	0.000	1.322	1.171–1.492	0.000	1.177	1.029–1.346	0.017
	RAR1	ref.	ref.	ref.	ref.	ref.	ref.	ref.	ref.	ref.
	RAR2	1.247	0.900–1.727	0.184	1.206	0.871–1.670	0.260	1.176	0.846–1.635	0.336
	RAR3	2.151	1.593–2.904	0.000	1.954	1.444–2.645	0.000	1.503	1.086–2.080	0.014
MACEs^3^										
	RAR^4^	1.235	1.125–1.355	0.000	1.196	1.086–1.317	0.000	1.128	1.017–1.252	0.023
	RAR1	ref.	ref.	ref.	ref.	ref.	ref.	ref.	ref.	ref.
	RAR2	1.389	1.106–1.745	0.005	1.346	1.072–1.691	0.011	1.320	1.048–1.664	0.019
	RAR3	1.768	1.415–2.210	0.000	1.624	1.298–2.033	0.000	1.452	1.141–1.846	0.002

Tablenotes: Model 1: Not adjusted. Model 2: Adjusted for gender and age. ^1^Model 3 for all-cause mortality, ^2^Model 3 for cardiovascular mortality, ^3^Model 3 for MACEs: adjusted for anemia, gender, hypertension, age, dialysis modality, left main disease, vintage, cause of dialysis, heart failure, smoking, multi-vessel disease, alkaline phosphatase, percutaneous coronary intervention (PCI), radial access, ARB or ACE inhibitor, index presentation. ^4^RAR was analyzed as a continuous variable. HR, hazard ratio; CI, confidence interval; MACEs, major adverse cardiovascular events; ref., reference.

When RAR was modeled as a continuous variable, a remarkably positive link was observed between elevated RARs and the risks of all-cause mortality (HR: 1.229, 95% CI: 1.101–1.372), cardiovascular mortality (HR: 1.177, 95% CI: 1.029–1.346), and MACEs (HR: 1.128, 95% CI: 1.017–1.252). These links were similarly significant when examining non-fatal myocardial infarction and non-fatal stroke (**Supplementary Table 5**).

**Fig. 4.**
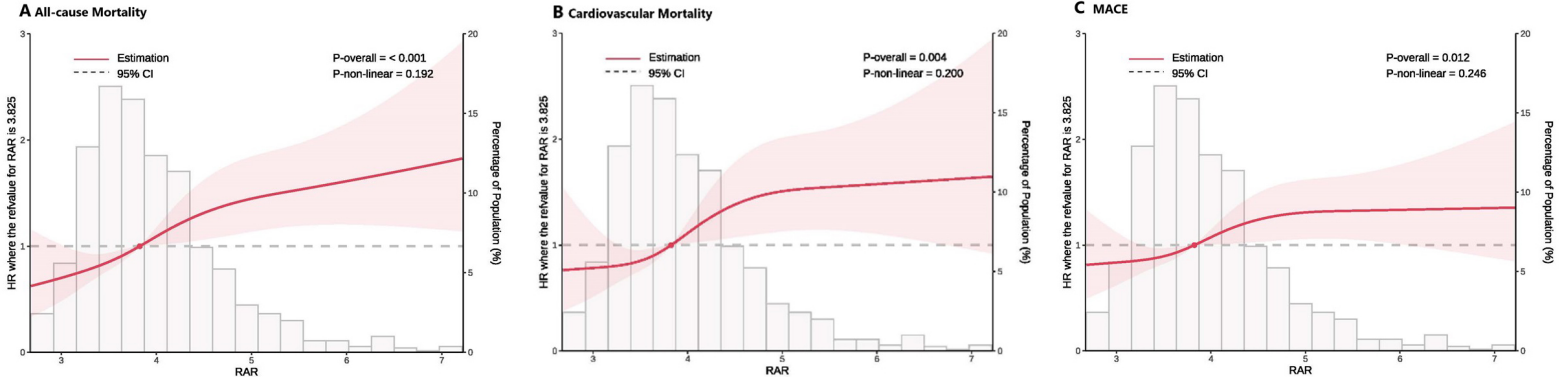
**Multivariable RCS regression analysis**. Linear association between RAR and primary and key secondary outcomes after full adjustment (Model 3). (A) Association of the RAR with all-cause mortality. (B) Association of the RAR with cardiovascular mortality. (C) Association of the RAR with MACEs. RCS, restricted cubic splines.

The RCS analysis revealed a clear, linear association between RAR levels and clinical outcomes, such as all-cause mortality, cardiovascular mortality, and MACEs. As RAR increased, the risk for these outcomes was progressively elevated, which was consistent across all subgroups analyzed. This linear relationship remained statistically significant even after adjusting for confounders (*p* < 0.05), enhancing the predictive value of the RAR for adverse clinical outcomes (*p* < 0.05; Fig. 4).

### 3.3 Increased Prognostic Utility of RAR Beyond Gensini and GRACE Scores

The integration of the RAR significantly raised the predictive performance of both Gensini and GRACE models, particularly for all-cause mortality (Table 3). Specifically, after incorporation of RAR into the GRACE model, a remarkable improvement was noted in both reclassification and discriminatory power, as evidenced by an IDI of 0.012 (*p* = 0.001; 95% CI: 0.005–0.019, etc.) and an NRI of 0.251 (*p* < 0.001; 95% CI: 0.129–0.373). Similarly, addition of RAR to the Gensini model resulted in a comparable enhancement, with the IDI improving to 0.021 (*p* < 0.001; 95% CI: 0.012–0.030) and the NRI elevating to 0.232 (*p* < 0.001; 95% CI: 0.111–0.354).

**Table 3.** **Improved predictive accuracy and reclassification statistics of the RAR concerning all-cause and cardiovascular mortality**.

	Model	NRI	*p*	IDI	*p*
All-cause mortality	GRACE	ref.	ref.	ref.	ref.
	GRACE+RAR	0.251 (0.129–0.373)	<0.001	0.012 (0.005–0.019)	0.001
	Gensini	ref.	ref.	ref.	ref.
	Gensini+RAR	0.232 (0.111–0.354)	<0.001	0.021 (0.012–0.030)	<0.001
Cardiovascular mortality	GRACE	ref.	ref.	ref.	ref.
	GRACE+RAR	0.330 (0.193–0.467)	<0.001	0.013 (0.005–0.020)	0.001
	Gensini	ref.	ref.	ref.	ref.
	Gensini+RAR	0.227 (0.090–0.364)	0.001	0.010 (0.004–0.017)	0.003

Tablenotes: NRI, net reclassification improvement; IDI, integrated discrimination improvement; GRACE, Global Registry of Acute Coronary Events; RAR, red cell distribution width-to-albumin ratio.

Incorporation of RAR also demonstrated a noticeable improvement in the predictive performance for cardiovascular mortality in both risk models (Table 3). For the GRACE model, the addition of the RAR resulted in an IDI of 0.013 (*p* = 0.001; 95% CI: 0.005–0.020) and an NRI of 0.330 (*p* <0.001; 95% CI: 0.193–0.467). When the RAR was included in the Gensini model, the IDI reached 0.010 (*p* = 0.003; 95% CI: 0.004–0.017), and the NRI was 0.227 (*p* = 0.001; 95% CI: 0.090–0.364).

For MACEs, inclusion of RAR improved the predictive performance of the Gensini model, whereas no significant improvement was observed when RAR was added to the GRACE model (**Supplementary Table 6**).

### 3.4 Subgroup Analysis

Subgroup analysis indicated a consistently strong association between RAR and all-cause mortality, cardiovascular mortality, and MACEs across a variety of patient subgroups, including those stratified by LVEF, age, atrial fibrillation, sex, reason for dialysis, anemia, percutaneous coronary intervention, Gensini score, and calcification severity. No statistically significant interactions were observed across these subgroups (all interaction *p* values > 0.05).

**Fig. 5.**
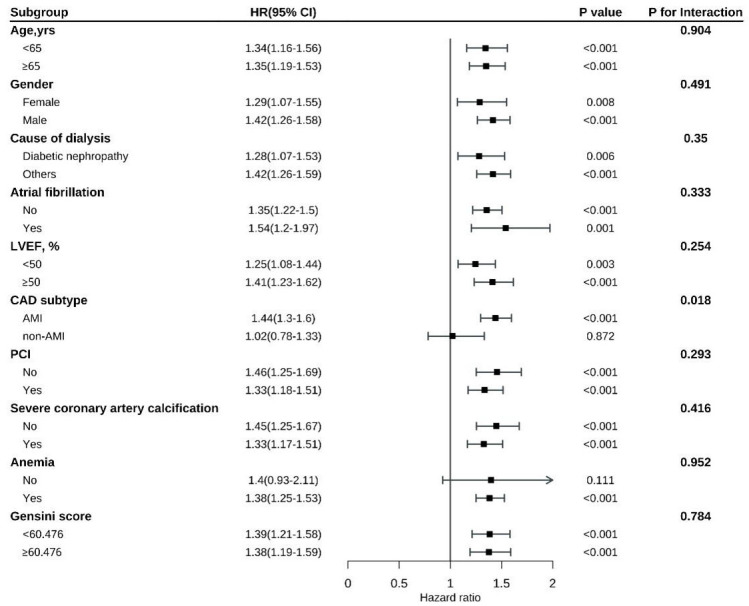
**Subgroup analysis of all-cause mortality by patient characteristics**.

**Fig. 6.**
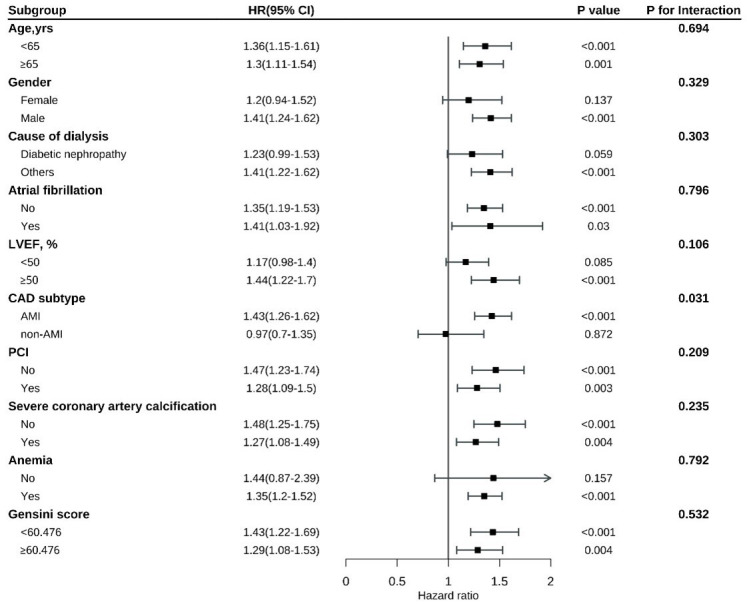
**Subgroup analysis of cardiovascular mortality across different patient characteristics**.

**Fig. 7.**
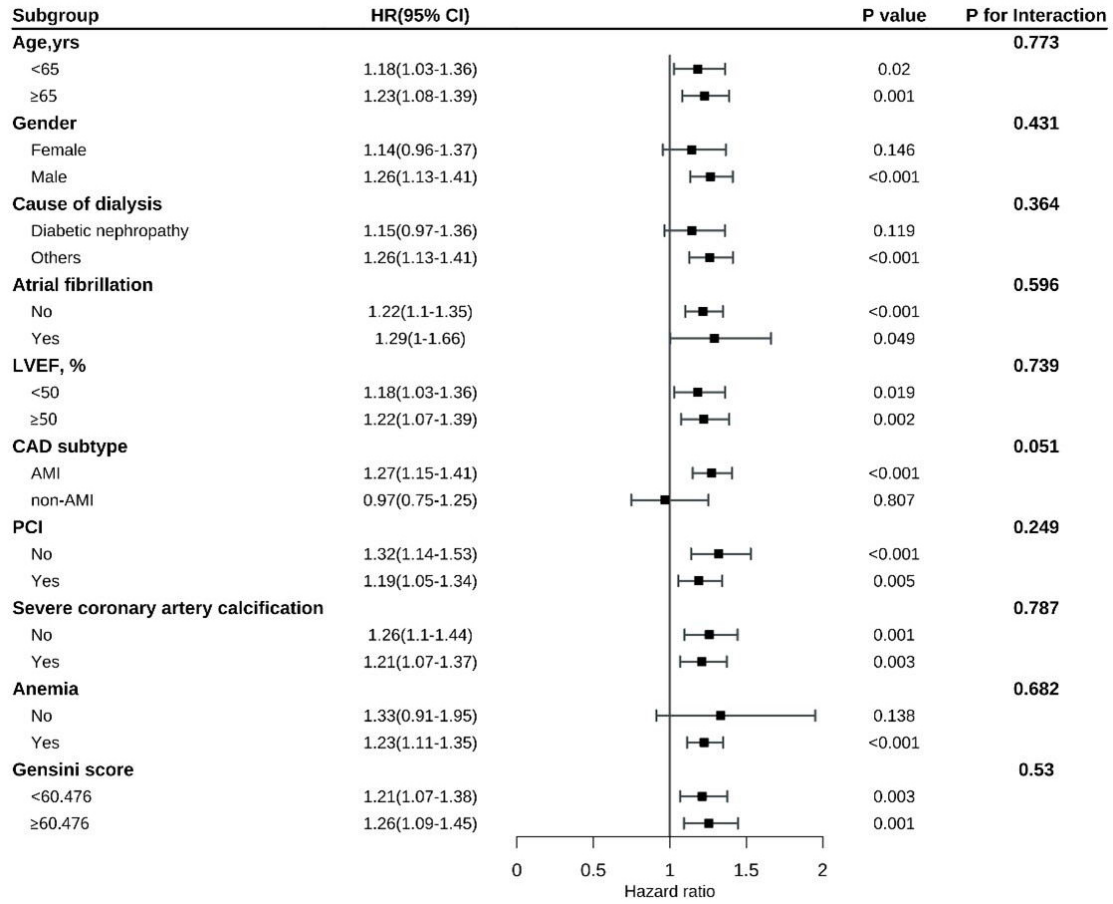
**Subgroup analysis of MACEs**.

In contrast, a significant interaction was identified between CAD subtype and both all-cause mortality (*p* = 0.018) and cardiovascular mortality (*p* = 0.031). Specifically, the association between a higher RAR and mortality risk was stronger in patients with acute myocardial infarction (AMI) than in those with stable angina (Figs. 5,6). The interaction between CAD subtype and the RAR for MACEs was marginally significant (*p* = 0.051; Fig. 7). These findings indicate that while the RAR remains a strong predictor, its predictive strength may vary slightly across CAD subtypes.

The adjustments made to the RDW detection method and the reference range specific to the dialysis cohort were confirmed to have no significant impact on the overall associations between RDW-related parameters and clinical outcomes. Sensitivity analyses were performed to assess the influence of dialysis-related factors, such as fluid shifts, the use of erythropoiesis-stimulating agents, and other dialysis-related variables, on RDW values. The results of these sensitivity analyses (Table 4) indicated that variations in RDW due to dialysis-related factors did not substantially alter the strength or direction of the associations between RDW and clinical outcomes. Specifically, the *p*-values for the sensitivity analyses comparing adjusted and unadjusted RDW parameters were not significant (*p* > 0.05), further confirming that these factors were effectively controlled.

**Table 4.** **Sensitivity analysis of RDW and dialysis-related factors on clinical outcomes**.

Sensitivity factor	Unadjusted RDW (hazard ratio)	Adjusted RDW (hazard ratio)	Change in hazard ratio	*p*-value
Dialysis modality (hemodialysis vs. peritoneal)	1.302 (1.138–1.500)	1.294 (1.120–1.485)	No significant change	0.479
Vintage (years)	1.220 (1.087–1.364)	1.215 (1.080–1.355)	No significant change	0.520
Diabetes mellitus	1.379 (1.254–1.517)	1.368 (1.244–1.498)	No significant change	0.532
Cause of dialysis (diabetes vs. others)	1.246 (1.114–1.392)	1.233 (1.106–1.370)	No significant change	0.681
Albumin fluctuation (pre- vs. post-dialysis)	1.316 (1.198–1.448)	1.309 (1.187–1.432)	No significant change	0.513
RDW-CV as a continuous variable	1.384 (1.257–1.523)	1.336 (1.209–1.477)	Slight reduction	0.000
Cardiovascular mortality (Model 3 adjusted)	1.459 (1.352–1.576)	1.422 (1.312–1.528)	Slight reduction	0.003
All-cause mortality (Model 3 adjusted)	1.592 (1.485–1.702)	1.572 (1.468–1.682)	Slight reduction	0.002
MACEs (Model 3 adjusted)	1.624 (1.498–1.759)	1.614 (1.488–1.745)	Slight reduction	0.004
Glucose levels (pre- vs. post-dialysis adjusted)	1.132 (1.012–1.268)	1.124 (1.003–1.261)	No significant change	0.382

Tablenotes: RDW, red blood cell distribution width.

## 4. Discussion

To our knowledge, the CRUISE study has been the largest retrospective cohort to date investigating the prognostic utility of the RAR in CAD patients receiving dialysis. The outcomes of the current investigation indicated a strong association between the highest RAR tertile and increased risks of all-cause mortality, cardiovascular mortality, and MACEs. Multivariate Cox proportional hazards modeling further confirmed that elevated RARs independently predicted these adverse clinical outcomes. Additionally, RCS analysis revealed a linear association between RAR and the risk of these endpoints, emphasizing the strength and consistency of the RAR as a prognostic biomarker. Moreover, integrating RAR into the Gensini and GRACE scoring models notably improved their predictive accuracy for mortality outcomes, reflecting the utility of the RAR as an adjunct to established risk stratification tools.

The RDW, a routinely assessed hematological parameter, serves as an indicator of RBC volume heterogeneity. RDW elevation in dialysis-dependent chronic kidney disease (CKD) patients can be attributed to multifactorial processes, most notably systemic inflammation and malnutrition, both of which are prevalent in this population. Uremic toxins, exposure to dialysate, and access-related infections act as major contributors to these conditions [14]. Inflammatory cytokines and oxidative stress disrupt erythropoiesis, resulting in an elevation in the proportion of immature RBCs, thereby escalating RDW. Additionally, lipid peroxidation impairs RBC deformability and further elevates RDW [15, 16]. When RDW exceeds a threshold of 14%, it impairs the efficiency of oxygen transport, enhances blood viscosity, and exacerbates the risk of myocardial ischemia and infarction, thereby contributing to adverse cardiovascular events [17].

Chronic systemic inflammation, a hallmark of dialysis-dependent CKD, is also responsible for appetite suppression and impaired nutrient intake via pro-inflammatory cytokines, resulting in hypoalbuminemia and protein-energy wasting [18]. Albumin, a negative acute-phase reactant with significant antioxidant and anti-inflammatory properties, has a remarkable function in maintaining vascular integrity and modulating inflammatory responses. Low serum albumin levels exhibited a notable association with CAD progression and serve as a marker of poor cardiovascular outcomes in this patient population [19, 20, 21, 22]. Consistently, the current study showed that patients in the highest RAR tertile exhibited both elevated RDW and diminished albumin levels, reflecting the multifactorial nature of these biomarkers in CAD pathogenesis in ESRD.

The RAR, a composite inflammatory index, may provide a more comprehensive reflection of the systemic inflammatory burden than either RDW or albumin alone. This index has demonstrated significant prognostic value in various cardiovascular/cerebrovascular diseases [23, 24]. Notably, Kimura *et al*. [13] pointed out that a high RAR was predictive of the progression to ESRD, as well as of increased mortality and enhanced frequency of cardiovascular events in CKD patients. Similarly, studies analyzing the MIMIC-III database and intensive care unit cohorts have identified the RAR as an independent predictor of mortality (i.e., short-/long-term) [25, 26]. Consistently, Liu *et al*. [27] found that the RAR was strongly associated with an elevated risk of cardiovascular disease. The higher the RAR, the greater the all-cause mortality and cardiovascular mortality rates [27].

Notably, we observed that the highest RAR tertile (Tertile 3) comprised a greater proportion of patients receiving peritoneal dialysis relative to hemodialysis. The function of dialysis modality in modulating the risk of CAD remains an area of active investigation and debate. Hemodialysis can induce a variety of pathophysiological alterations, involving volume shifts, blood pressure fluctuations, myocardial stunning, and electrolyte imbalances, all of which have the potential to exacerbate CAD and escalate arrhythmic risk [28]. On the other hand, the glucose-rich dialysate used in peritoneal dialysis may lead to accumulation of advanced glycation end-products, metabolic syndrome, and insulin resistance, all of which are known to promote atherogenesis and increase the risk of cardiovascular events. However, to date, no definitive evidence has conclusively favored one dialysis modality over another in terms of reducing the incidence of CAD or boosting cardiovascular outcomes in this patient population [29, 30].

Subgroup analysis indicated a potential interaction between RAR and clinical outcomes in AMI vs. non-AMI patients, with a more pronounced association observed among patients with acute myocardial infarction. This differential prognostic impact likely reflects the augmented systemic and local inflammatory response observed in AMI, which activates intricate cellular death pathways, including apoptosis, ferroptosis, and autophagy. The enhanced prognostic value of the RAR in the AMI subgroup is in concordance with previous studies that have highlighted its predictive significance in patients facing acute cardiovascular events [19, 25, 26].

As a composite inflammatory index, RAR offers a more holistic view on systemic inflammation and nutritional status, potentially overcoming the limitations of RDW and albumin when used in isolation. Future studies should investigate the underlying biological mechanisms, particularly in the context of dialysis-dependent patients. The pathophysiology of dialysis, characterized by chronic inflammation, oxidative stress, and uremic toxin accumulation, likely influences the relationship between RAR and adverse cardiovascular outcomes. A deeper exploration into how RAR interacts with these factors can provide insights into why it functions as a robust predictor of mortality and cardiovascular events in this unique population. Moreover, examining interactions of the RAR with dialysis modalities (e.g., hemodialysis vs. peritoneal dialysis) will help identify whether its prognostic value varies according to the type of renal replacement therapy.

This study has several strengths and limitations. A key strength is its focus on the association between the RAR and long-term outcomes in dialysis patients with CAD. We have integrated an extensive range of inpatient data, as well as the GRACE and Gensini scores, all of which are crucial for accurate risk stratification in this high-risk cohort. However, several limitations warrant consideration. First, the retrospective nature inherently raises concerns regarding confounding factors and potential selection bias, which could affect the robustness of the findings. The reliance on historical medical records limits our ability to control for unknown or unmeasured variables, thus introducing an element of uncertainty. Additionally, the retrospective design may limit the ability to account for evolving confounders, such as fluctuations in the RAR over time or changes in treatment protocols during follow-up, especially post-discharge medication adherence. Longitudinal data collection may enable a more comprehensive understanding of how the RAR changes with disease progression and how it interacts with other clinical and laboratory factors over time. Therefore, the results of this study should be interpreted with caution, and prospective studies are recommended to validate the findings and assess the robustness of the RAR as a prognostic marker in dialysis patients with CAD. Second, although the RDW is widely accepted in clinical practice to assess anemia, our study lacked data on other principal nutritional and hematological parameters, including folate, iron, and vitamin B12, which may influence the RAR and provide additional insights into mechanistic pathways. The absence of these variables may undervalue the multifactorial nature of the prognostic potential of the RAR. Third, we focused exclusively on baseline RAR values measured at the time of hospital admission, neglecting the potential prognostic implications of longitudinal fluctuations in the RAR over the course of dialysis treatment. Longitudinal assessments of the RAR may shed light on its dynamics as a biomarker for disease progression and response to therapeutic interventions. Future research integrating serial RAR measurements alongside time-dependent analysis will be essential for delineating the full prognostic capacity of the RAR. Fourth, CAD severity in this cohort was determined using the Gensini score. This approach was efficient in assessing the extent of coronary artery involvement; however, it may have lacked the granularity provided by other scoring systems (e.g., the SYNTAX score). The SYNTAX score, exhibiting a more comprehensive assessment of lesion complexity and vascular anatomy, may provide a more detailed perspective on CAD severity and its long-term prognostic impact. Future studies incorporating both the Gensini and SYNTAX scores will enable a more detailed assessment of CAD complexity and its influence on clinical outcomes in this patient population. Future prospective studies are essential to further confirm the prognostic value of the RAR in dialysis patients with CAD. These studies should aim to collect data from a more diverse, multi-center cohort and evaluate how RAR dynamics (as opposed to static baseline measures) influence long-term outcomes. Validation in other populations, including those undergoing different forms of renal replacement therapy, will be crucial for assessing the generalizability of the predictive power of the RAR. Additionally, large-scale prospective studies may help address potential selection biases inherent to retrospective analyses.

## 5. Conclusions

Our study provides compelling evidence that an increased RAR is independently associated with an elevated risk of all-cause mortality and cardiovascular mortality in dialysis-dependent patients with CAD. The integration of the RAR into the Gensini and GRACE risk models remarkably amplified their predictive accuracy, reflecting the utility of the RAR as a potent biomarker for prognosis in this high-risk population. Given its widespread availability, simplicity, and strong prognostic value, the RAR has emerged as a promising tool for routine clinical usage in evaluating dialysis patients with CAD. It holds noticeable potential for improving risk stratification, guiding clinical decision-making, and ultimately enhancing patient outcomes in this vulnerable cohort.

## Data Availability

The datasets involved in the current study (CRUISE-R) are unavailable publicly owing to restrictions on data sharing imposed by ethical considerations, patient confidentiality agreements, and institutional policies. However, access to these data may be granted upon formal request to the corresponding author, subject to reasonable terms and conditions, contingent upon adherence to appropriate ethical review and approval processes, and subject to necessary data privacy and security protocols.

## Abbreviations

AIC, Akaike Information Criterion; AMI, Acute Myocardial Infarction; ANOVA, Analysis of Variance; BIC, Bayesian Information Criterion; CABG, Coronary Artery Bypass Grafting; CAD, Coronary Artery Disease; CI, Confidence Interval; CRUISE-R, Coronary Revascularization in Patients on Dialysis in China - Retrospective; CKD, Chronic Kidney Disease; eGFR, Estimated Glomerular Filtration Rate; ESRD, End-Stage Renal Disease; GRACE, Global Registry of Acute Coronary Events; HDL-C, High-Density Lipoprotein Cholesterol; HR, Hazard Ratio; IDI, Integrated Discrimination Improvement; IQR, Interquartile Range; LVEF, Left Ventricular Ejection Fraction; MACEs, Major Adverse Cardiovascular Event(s); NRI, Net Reclassification Improvement; PCI, Percutaneous Coronary Intervention; RAR, Red cell distribution width-to-Albumin Ratio; RBC, Red Blood Cell; RCS, Restricted Cubic Spline; RDW, Red Blood Cell Distribution Width; SD, Standard Deviation; STROBE, Strengthening the Reporting of Observational Studies in Epidemiology.

## Supplementary Material

Supplementary material associated with this article can be found, in the online version, at https://doi.org/10.31083/RCM47939.
