# Supplementary material for: A New and Robust Prognostic Biomarker for Mortality Risk Prediction in Dialysis Patients With Coronary Artery Disease: Red Cell Distribution Width-to-Albumin Ratio
Source: Rev Cardiovasc Med. 2026 Apr 20;27(4):47939. doi: 10.31083/RCM47939 (PMC13155975; doi:10.31083/RCM47939)
Supplement: Supplementary file 1 [file 2153-8174-27-4-47939-s1.zip › Supplementary Material.docx]

**SUPPLEMENTAL MATERIAL**

**Table S1. List of Principal Investigators and Participating Centers**

| **No.** | **Name** | **Affiliation** | **Region** | **Province** |
| --- | --- | --- | --- | --- |
| 1 | Jingang Zheng | China-Japan Friendship Hospital | North China | Beijing |
| 2 | Yong He | West China Hospital | Southwest China | Sichuan |
| 3 | Hesong Zeng | Tongji Hospital | Central China | Hubei |
| 4 | Jianfang Luo | Guangdong Provincial People’s Hospital | South China | Guangdong |
| 5 | Mulei Chen | Beijing Chaoyang Hospital | North China | Beijing |
| 6 | Wenyue Pang | Shengjing Hospital of China Medical University | Northeast China | Liaoning |
| 7 | Yanmin Xu | Second Hospital of Tianjin Medical University | North China | Tianjin |
| 8 | Chuanyu Gao | Fuwai Central China Cardiovascular Hospital | Central China | Henan |
| 9 | Xiaogang Guo | The First Affiliated Hospital, Zhejiang University School of Medicine | East China | Zhejiang |
| 10 | Lin Cai | The Third People's Hospital of Chengdu | Southwest China | Sichuan |
| 11 | Qingwei Ji | The People’s Hospital of Guangxi Zhuang Autonouous Region | South China | Guangxi Zhuang Autonomous Region |
| 12 | Yining Yang | People's Hospital of Xinjiang Uygur Autonomous Region | Northwest China | Xinjiang |
| 13 | Di Wu | Emgency General Hospital | North China | Beijing |
| 14 | Yiqiang Yuan | Henan Provincial Chest Hospital | Central China | Henan |
| 15 | Jing Wan | Zhongnan Hospital of Wuhan University | Central China | Hubei |
| 16 | Yuliang Ma | Peking university People’s hospital | North China | Beijing |
| 17 | Jun Zhang | Cangzhou Central Hospital of Tianjin Medical University | North China | Hebei |
| 18 | Zhimin Du | Dongguan Tungwah Hospital | South China | Guangdong |
| 19 | Qing Yang | Tianjin Medical University General Hospital | North China | Tianjin |
| 20 | Jinsong Cheng | Ningbo First Hospital | East China | Zhejiang |
| 21 | Chunhua Ding | Aerospace Center Hospital | North China | Beijing |
| 22 | Xiang Ma | The First Affiliated Hospital of Xinjiang Medical University | Northwest China | Xinjiang |
| 23 | Chunlin Yin | Xuanwu Hospital | North China | Beijing |
| 24 | Zeyuan Fan | Civil Aviation General Hospital | North China | Beijing |
| 25 | Qiang Tang | Peking University Shougang Hospital | North China | Beijing |
| 26 | Yue Li | the First Affiliated Hospital, Harbin Medical University | Northeast China | Heilongjiang |
| 27 | Lihua Sun | Fifth Affiliated Hospital of Xinjiang Medical University | Northwest China | Xinjiang |
| 28 | Chengzhi Lu | First Central Hospital of Tianjin | North China | Tianjin |
| 29 | Jufang Chi | Shaoxing People's Hospital | East China | Zhejiang |
| 30 | Zhuhua Yao | Tianjin Union Medicine Center | North China | Tianjin |

**Table S2. Definitions of GRACE score and Gensini score**

**S2.1 GRACE Score**

Below is the **GRACE Score Table** for predicting the 30-day mortality risk in patients with acute coronary syndrome (ACS). It includes all of the key variables used in calculating the GRACE score, with each factor having its corresponding points based on patient values^[1]^.

| **Variable** | **Score Range** | **Points Assigned** |
| --- | --- | --- |
| Age (years) | 18-39 | 0-27 points |
|  | 40-49 | 0-24 points |
|  | 50-59 | 0-21 points |
|  | 60-69 | 0-17 points |
|  | 70-79 | 0-12 points |
|  | 80+ | 0-7 points |
| Heart Rate (bpm) | <60 | 0 points |
|  | 60-79 | 10 points |
|  | 80-89 | 12 points |
|  | 90-99 | 14 points |
|  | 100-109 | 16 points |
|  | ≥110 | 18 points |
| Systolic BP (mmHg) | <70 | 10 points |
|  | 70-79 | 6 points |
|  | 80-89 | 4 points |
|  | 90-99 | 2 points |
|  | 100-109 | 0 points |
|  | ≥110 | -2 points |
| Serum Creatinine (mg/dL) | <1.0 | 0 points |
|  | 1.0-1.9 | 6 points |
|  | ≥2.0 | 12 points |
| Killip Class | I (no heart failure) | 0 points |
|  | II (mild heart failure) | 8 points |
|  | III (acute pulmonary edema) | 17 points |
|  | IV (cardiogenic shock) | 23 points |
| Cardiac Arrest at Admission | Yes | 17 points |
|  | No | 0 points |
| ST Segment Deviation | No deviation | 0 points |
|  | ST depression | 8 points |
|  | ST elevation | 17 points |
| Troponin (if available) | Normal | 0 points |
|  | Elevated | 10 points |

To calculate the GRACE score, sum up the points from all the relevant variables based on the patient’s clinical data. Once you have the total score, use it to determine the patient’s risk category.

2.1.1 Risk Categories:

Low Risk: GRACE score ≤ 140 points

Moderate Risk: GRACE score 141-220 points

High Risk: GRACE score > 220 points

2.1.2 30-Day Mortality Risk:

Low Risk: < 1% mortality

Moderate Risk: 1-5% mortality

High Risk: > 5% mortality

**S2.2 Gensini Score**

The **Gensini Score** is a clinical tool used to assess the severity of coronary artery disease (CAD) based on coronary angiography. The Gensini score takes into account both the degree of coronary stenosis (narrowing) and the location of the stenotic lesions. The score is widely used to evaluate the extent of coronary artery disease and to predict cardiovascular events and patient prognosis^[2]^.

**Gensini Scoring System:**

**2.2.1 Lesion location:** The coronary arteries are divided into different segments, and the score is assigned based on the location of the coronary artery lesion. The proximal coronary lesions (closer to the heart) are assigned a higher weight, reflecting their greater clinical significance.

- - **Left main coronary artery**: 5 points
  - **Proximal Left Anterior Descending (LAD)**: 4 points
  - **Proximal Left Circumflex (LCX)**: 4 points
  - **Proximal Right Coronary Artery (RCA)**: 3 points
  - **Mid LAD**: 3 points
  - **Mid LCX**: 3 points
  - **Mid RCA**: 2 points
  - **Distal LAD**: 2 points
  - **Distal LCX**: 2 points
  - **Distal RCA**: 1 point

**2.2.2 Degree of stenosis:** Points are assigned according to the degree of coronary artery narrowing or stenosis. The greater the narrowing, the higher the score.

- - 1% to 25% stenosis: 0 points
  - 26% to 50% stenosis: 1 point
  - 51% to 75% stenosis: 2 points
  - 76% to 90% stenosis: 3 points
  - 91% to 99% stenosis: 4 points
  - 100% occlusion: 5 points

**2.2.3 Scoring method:**

- - The score for each lesion is calculated by multiplying the lesion’s location score by the degree of stenosis.
  - For example, a lesion in the proximal LAD with 50% stenosis would score 4 (location) × 2 (degree of stenosis) = 8 points.
  - All lesion scores are added together to obtain the total Gensini score.

**2.2.4 Interpretation of Gensini Score:**

- - **Mild CAD**: 0 to 20 points
  - **Moderate CAD**: 21 to 40 points
  - **Severe CAD**: > 40 points

The Gensini score reflects both the extent and severity of coronary artery disease, and a higher score is typically associated with worse prognosis, including higher rates of mortality and cardiovascular events.

**Table S3. Definitions of events**

| **Event** | **Definition** |
| --- | --- |
| All-cause mortality | All-cause mortality was defined as any cause of death from inclusion until latest follow-up. |
| Cardiovascular mortality | Cardiovascular mortality was defined as death due to acute myocardial infarction, heart failure, sudden cardiac death, stroke, cardiovascular procedure, or cardiovascular hemorrhage. |
| Acute myocardial infarction (AMI) | The definition of acute myocardial infarction (AMI) was the presence of myocardial necrosis, with clinical manifestations, cardiac biomarkers, and imaging findings consistent with acute myocardial ischemia, in accordance with the third universal definition of myocardial infarction. |
| Acute stroke | The diagnostic criteria for acute stroke included a sudden onset of neurological deficits, attributable to a focal disruption in cerebral blood flow due to ischemia or hemorrhage, with symptoms lasting less or more than 24 hours. Diagnosis was confirmed through computed tomography (CT) or magnetic resonance imaging (MRI) to distinguish between hemorrhagic and ischemic strokes. |

**Table S4. The events of primary and secondary outcomes**

MI, myocardial infarction; MACE, Major Adverse Cardiovascular Events.

| Characteristic | Overall  N=1128 | Tertile1 (RAR≤3.56)  N=376 | Tertile 2 (3.56＜RAR≤4.13)  N=376 | Tertile 3 (RAR＞4.13)  N=376 | P value |
| --- | --- | --- | --- | --- | --- |
| All-cause mortality | 378 | 97 (25.7) | 119 (31.5) | 162 (42.8) | ＜0.001 |
| Cardiovascular mortality | 261 | 69 (26.4) | 77 (29.5) | 115 (44.1) | ＜0.001 |
| MACE | 485 | 135 | 165 | 185 | 0.001 |
| Non-fatal MI | 111 | 37 | 45 | 29 | 0.147 |
| Non-fatal stroke | 41 | 13 | 17 | 11 | 0.492 |

**Table S5. Associations between non-fatal MI and non-fatal stroke**

Model 1: Unadjusted. Model 2: Adjusted for age and gender

†Model 3 for non-fatal MI: Adjusted for age, gender, cause of dialysis, vintage, dialysis modality, hypertension, smoking, heart failure, anemia, alkaline phosphatase, left main disease, multi-vessel disease, radial access, PCI treatment, index presentation, GRACE score, ACE inhibitor or ARB.

‡Model 3 for non-fatal stroke: Adjusted for age, gender, cause of dialysis, vintage, dialysis modality, hypertension, smoking, heart failure, anemia, alkaline phosphatase, left main disease, multi-vessel disease, radial access, PCI treatment, index presentation, GRACE score, ACE inhibitor or ARB.

§RAR analyzed as a continuous variable.

MI, myocardial infarction.

|  | **Model 1** | | |  | **Model 2** | | |  | **Model 3** | | |
| --- | --- | --- | --- | --- | --- | --- | --- | --- | --- | --- | --- |
|  | HR | 95%CI | P |  | HR | 95%CI | P |  | HR | 95%CI | P |
| **Non-fatal MI†** | | | | | | | | | | | |
| RAR§ | 1.384 | 1.257-1.523 | 0.000 |  | 1.336 | 1.209-1.477 | 0.000 |  | 1.229 | 1.101-1.372 | 0.000 |
| RAR1 | ref. | ref. | ref. |  | ref. | ref. | ref. |  | ref. | ref. | ref. |
| RAR2 | 1.379 | 1.054-1.804 | 0.019 |  | 1.325 | 1.013-1.733 | 0.04 |  | 1.296 | 0.988-1.702 | 0.061 |
| RAR3 | 2.193 | 1.703-2.824 | 0.000 |  | 1.959 | 1.518-2.528 | 0.000 |  | 1.592 | 1.212-2.092 | 0.001 |
| **Non-fatal stroke‡** | | | | | | | | | | | |
| RAR§ | 1.362 | 1.212-1.531 | 0.000 |  | 1.322 | 1.171-1.492 | 0.000 |  | 1.177 | 1.029-1.346 | 0.017 |
| RAR1 | ref. | ref. | ref. |  | ref. | ref. | ref. |  | ref. | ref. | ref. |
| RAR2 | 1.247 | 0.900-1.727 | 0.184 |  | 1.206 | 0.871-1.670 | 0.260 |  | 1.176 | 0.846-1.635 | 0.336 |
| RAR3 | 2.151 | 1.593-2.904 | 0.000 |  | 1.954 | 1.444-2.645 | 0.000 |  | 1.503 | 1.086-2.080 | 0.014 |

**Table S6. Improved predictive accuracy and reclassification statistics of RAR concerning MACE.**

| **Model** | **NRI** | **P** | **IDI** | **P** |
| --- | --- | --- | --- | --- |
| **Grace** | ref. | ref. | ref. | ref. |
| **Grace+RAR** | 0.115(-0.001-0.232) | 0.052 | 0.002(0-0.005) | 0.094 |
| **Gensini** | ref. | ref. | ref. | ref. |
| **Gensini+RAR** | 0.087(-0.029-0.203) | 0.141 | 0.008(0.002-0.013) | 0.004 |

GRACE, global registry of acute coronary events; IDI, Integrated Discrimination Improvement; NRI, Net Reclassification Improvement; RAR, red cell distribution width to albumin ratio.

**References**

[1] Granger C B, Goldberg R J, Dabbous O, *et al*. Predictors of hospital mortality in the global registry of acute coronary events. Archives of internal medicine. 2003; 163(19): 2345-2353.

[2] Gensini G G. A more meaningful scoring system for determining the severity of coronary heart disease. Am J cardiol. 1983; 51: 606.
